# Brain disconnectome mapping derived from white matter lesions and serum neurofilament light levels in multiple sclerosis: A longitudinal multicenter study

**DOI:** 10.1016/j.nicl.2022.103099

**Published:** 2022-06-25

**Authors:** Henning H. Rise, Synne Brune, Claudia Chien, Tone Berge, Steffan D. Bos, Magí Andorrà, Irene Pulido Valdeolivas, Mona K. Beyer, Piotr Sowa, Michael Scheel, Alexander U. Brandt, Susanna Asseyer, Kaj Blennow, Mads L. Pedersen, Henrik Zetterberg, Michel Thiebaut de Schotten, Maria Cellerino, Antonio Uccelli, Friedemann Paul, Pablo Villoslada, Hanne F. Harbo, Lars T. Westlye, Einar A. Høgestøl

**Affiliations:** a NORMENT, Division of Mental Health and Addiction, Oslo University Hospital, Oslo, Norway; b Department of Psychology, University of Oslo, Oslo, Norway; c Institute of Clinical Medicine, University of Oslo, Oslo, Norway; d Department of Neurology, Oslo University Hospital, Oslo, Norway; e Charité –Universitätsmedizin Berlin, Corporate Member of Freie Universität Berlin and Humboldt-Universität zu Berlin & Max Delbrück Center for Molecular Medicine in the Helmholtz Association, Experimental and Clinical Research Center, Germany; f Charité –Universitätsmedizin Berlin, Corporate Member of Freie Universität Berlin and Humboldt-Universität zu Berlin, NeuroCure Clinical Research Center, Germany; g Charité –Universitätsmedizin Berlin, Corporate Member of Freie Universität Berlin, Humboldt-Universität zu Berlin, Department for Psychiatry and Psychotherapy, Germany; h Department of Mechanical, Electronics and Chemical Engineering, Oslo Metropolitan University, Oslo, Norway; i Department of Research, Innovation and Education, Oslo University Hospital, Oslo, Norway; j Institut d’Investigacions Biomèdiques August Pi Sunyer, Barcelona, Spain; k Division of Radiology and Nuclear Medicine, Oslo University Hospital, Oslo, Norway; l Charité –Universitätsmedizin Berlin, Corporate Member of Freie Universität Berlin, Humboldt-Universität zu Berlin, Department of Neuroradiology, Germany; m Department of Neurology, University of California, Irvine, CA, USA; n Clinical Neurochemistry Laboratory, Department of Psychiatry and Neurochemistry, Institute of Neuroscience and Physiology, Sahlgrenska Academy, University of Gothenburg, Mölndal, Sweden; o Department of Neurodegenerative Disease, Institute of Neurology, University College London, London, United Kingdom; p UK Dementia Research Institute at UCL, London, United Kingdom; q Brain Connectivity and Behaviour Laboratory, Sorbonne Universities, Paris, France; r Groupe d’Imagerie Neurofonctionnelle, Institut des Maladies Neurodégénératives- UMR 5293, CNRS, CEA University of Bordeaux, Bordeaux, France; s Department of Neurosciences, Rehabilitation, Ophthalmology, Genetics, Maternal and Child Health, University of Genoa, Genoa, Italy; t IRCCS Ospedale Policlinico San Martino, Genoa, Italy; u KG Jebsen, Centre for Neurodevelopmental Disorders, University of Oslo, Oslo, Norway

**Keywords:** **3D BRAVO**, 3D sagittal brain volume, **CAT12**, Computational Anatomy Toolbox for SPM12, **CIS**, clinically isolated syndrome, **CLSM**, connectome-based lesions symptom mapping, **DMT**, disease modifying treatments, **DTI**, diffusion tensor imaging, **EDSS**, Expanded Disability Status Scale, **FLAIR**, fluid-attenuated inversion recovery, **FSPGR**, fast-spoiled gradient-echo, **GA**, glatiramer acetate, **GD**, global disconnectivity, **GLM**, general linear model, **HC**, healthy controls, **ID**, identifier, **IFN**, interferon, **IQR**, Interquartile range, **MNI**, Montreal Neurological Institute, **MPRAGE**, magnetization prepared rapid gradient echo, **NfL**, neurofilament light chain, **PBVC**, percent brain tissue volume change, **PMS**, progressive multiple sclerosis, **PPMS**, primary progressive multiple sclerosis, **RIS**, radiologically isolated syndrome, **rLMM**, robust linear mixed-effects models, **RRMS**, remitting-remitting multiple sclerosis, **SIMOA**, single molecule array, **SPMS**, secondary progressive multiple sclerosis, **T**, Tesla, **T2LV**, **T2** lesion volume, **TE**, echo time, **TFCE**, threshold-free cluster enhancement, **TFE**, turbo field echo, **TR**, repetition time, Multiple sclerosis, Magnetic resonance imaging, Neurofilament, Disconnectome mapping, Longitudinal

## Abstract

**Background and Objectives:**

Connectivity-based approaches incorporating the distribution and magnitude of the extended brain network aberrations caused by lesions may offer higher sensitivity for axonal damage in patients with multiple sclerosis (MS) than conventional lesion characteristics. Using individual brain disconnectome mapping, we tested the longitudinal associations between putative imaging-based brain network aberrations and levels of serum neurofilament light chain (NfL) as a neuroaxonal injury biomarker.

**Methods:**

MS patients (n = 312, mean age 42.9 years, 71 % female) and healthy controls (HC) (n = 59, mean age 39.9 years, 78 % female) were prospectively enrolled at four European MS centres, and reassessed after two years (MS, n = 242; HC, n = 30). Post-processing of 3 Tesla (3 T) MRI data was performed at one centre using a harmonized pipeline, and disconnectome maps were calculated using BCBtoolkit based on individual lesion maps. Global disconnectivity (GD) was defined as the average disconnectome probability in each patient’s white matter. Serum NfL concentrations were measured by single molecule array (Simoa). Robust linear mixed models (rLMM) with GD or T2-lesion volume (T2LV) as dependent variables, patient as a random factor, serum NfL, age, sex, timepoint for visit, diagnosis, treatment, and center as fixed factors were run.

**Results:**

rLMM revealed significant associations between GD and serum NfL (t = 2.94, p = 0.003), age (t = 4.21, p = 2.5 × 10^−5^), and longitudinal changes in NfL (t = -2.29, p = 0.02), but not for sex (t = 0.63, p = 0.53) or treatments (t = 0.80–0.83, p = 0.41–0.42). Voxel-wise analyses revealed significant associations between dysconnectivity in cerebellar and brainstem regions and serum NfL (t = 7.03, p < 0.001).

**Discussion:**

In our prospective multi-site MS cohort, rLMMs demonstrated that the extent of global and regional brain disconnectivity is sensitive to a systemic biomarker of axonal damage, serum NfL, in patients with MS. These findings provide a neuroaxonal correlate of advanced disconnectome mapping and provide a platform for further investigations of the functional and potential clinical relevance of brain disconnectome mapping in patients with brain disorders.

## Highlights

• Individual disconnectome maps generated using a template of 7T MRI data.
• Disconnectome maps conceptualize distal brain network aberrations.
• Using lesions maps from our MS cohort, we produced individual disconnectome maps.
• Serum neurofilament light levels were associated with disconnectome maps.
• Voxel-wise analyses revealed interesting association with serum neurofilament light levels.

## 1 Introduction

MRI of the brain and spinal cord is essential for diagnostics and clinical management of patients with multiple sclerosis (MS), a chronic autoimmune disorder of the CNS (Thompson et al., 2018). Despite recent advances, conventional MRI markers typically show modest associations with clinical measures of disease progress and disability (Filippi et al., 2020). MRI technology improvements have enabled highly accurate visualization of white matter lesions due to MS-related pathological processes (Filippi et al., 2016, Geraldes et al., 2018). While T2-weighted lesions represent important MRI markers in MS, their neuroanatomical and pathophysiological implications are complex, and their predictive value for clinical trajectories and outcomes has been modest (Filippi et al., 2019, Filippi et al., 2016, Geraldes et al., 2018). This clinico-radiological-paradox may be alleviated by imaging methods sensitive to subtle primary or secondary axonal damage extending beyond the visual foci of the lesions (Barkhof, 2002, Mollison et al., 2017). Previous research has shown that MRI features probing physical and microstructural properties of the brain may provide sensitive markers of demyelination and axonal damage in MS (Chamberland et al., 2020, Rocca et al., 2015). Especially in advanced medical imaging, high-resolution diffusion MRI has been promising to evaluate brain disconnections resulting from white matter lesions (Chamberland et al., 2020).

Recent advances in brain imaging techniques probing the human brain connectome have allowed for in vivo investigations of the distributed network-level aberrations caused by focal lesions (Thiebaut de Schotten et al., 2020). By calculating the probability that white matter fibers, as identified in healthy individuals, intersect lesions observed in the brain of MS patients, individual disconnectome maps offer a conceptualization and characterization of the extent of brain network aberrations due to local T2-hyperintense lesions.

Simpler models of connectome modelling termed connectome-based lesions symptom mapping (CLSM) have shown promise for predicting future processing speed in subjects with MS (Gleichgerrcht et al., 2017, Kuceyeski et al., 2018). While previous studies have used both functional MRI (Meijer et al., 2020) and diffusion weighted imaging (Gleichgerrcht et al., 2017), the neurobiological and pathological correlates of disconnectome mapping remain unknown, partly due to the non-invasive nature of the procedures and the lack of accurate biomarkers reflecting axonal damage.

Over the last two decades, central (CSF) and peripheral (plasma and serum) neurofilaments have gained increased attention as candidate biomarkers of neuroaxonal injury as these structural scaffolding proteins are exclusively expressed in neurons and released into the periphery upon axonal damage with potential to monitor subclinical disease activity in neurodegenerative disorders such as MS. Neurofilament light chain (NfL) is a subunit of neurofilaments, and the concentration levels of neurofilament protein in CSF and blood has been shown to increase proportionally to the degree of axonal damage (Gaetani et al., 2019). Recent studies employing the single molecule array (Simoa®) technique have demonstrated that NfL concentrations in serum and CSF are highly correlated, enabling the use of serum NfL as a reliable biomarker reflecting axonal injury (Disanto et al., 2017, Kuhle et al., 2016). Higher levels of serum or CSF NfL have been associated with MRI lesions, spinal cord and brain atrophy, gadolinium-enhancing lesions, increasing age, recent clinical relapses, clinical disability, and treatment efficacy in MS (Barro et al., 2018, Benkert et al., 2022, Brune et al., 2022, Disanto et al., 2017, Gunnarsson et al., 2011, Hakansson et al., 2017, Ineichen et al., 2021, Piehl et al., 2018, Preziosa et al., 2020, Tavazzi et al., 2020, Varhaug et al., 2018). Recent studies based on national health registries proposed NfL as a potential marker for predicting MS risk and disease course, even at the earlier stages of the disease in subjects with radiologically isolated syndrome (RIS) and clinically isolated syndrome (CIS) (Bjornevik et al., 2020, Preziosa et al., 2020). Thus, combining NfL with advanced neuroimaging measures may enable increased understanding of MS pathophysiology and improved prognosis prediction as well as treatment responses monitoring (Bridel et al., 2021, Ineichen et al., 2021, Joisten et al., 2021, Preziosa et al., 2020, Saraste et al., 2021). In theory, the part of the axon distal to a white matter lesion due to MS will potentially be decaying as a primary or secondary consequence of the tissue damage, which then might result in various levels of higher NfL concentrations for an unknown period of time.

In this longitudinal multicenter study comprising a large real-world heterogeneous MS cohort (*n* = 312), we wanted to test if global brain disconnectivity could offer higher sensitivity to serum NfL concentrations as compared with conventional T2 lesion volumes (T2LV). To leverage the combined cross-sectional and longitudinal study design, we used robust linear mixed-effects models (rLMM) including relevant covariates to test for overall associations between global disconnectivity and serum NfL concentrations as well as the interactions between serum NfL concentration and changes in serum NfL on global disconnectivity. Subsequently, we performed voxel-wise analyses to map the neuroanatomical distribution of associations and to compare global disconnectivity with conventional measures, employing similar rLMM testing for associations with T2LV.

## 2 Materials and methods

### 2.1 Study population

A total of 328 MS patients were prospectively enrolled at four European MS centres from July 2016 to December 2017 (68 subjects from Hospital Clinic of Barcelona, Spain; 95 subjects from Oslo University Hospital, Norway; 73 subjects from Charité-Universitaetsmedizin Berlin, Germany; 92 subjects from Ospedale Policlinico San Martino, Genoa, Italy). All MS patients were invited for a two-year follow-up between January 2017 and March 2020, resulting in 280 subjects (85 %) completing the longitudinal study. For our study, 312 MS patients (95 %) met all criteria with available clinical, MRI, and serum NfL data at baseline, while 242 MS patients (86 %) fulfilled the requirements at follow-up (Table 1). All patients provided demographic information, personal MS history, blood samples, and assessment of Expanded Disability StatusScale (EDSS).

**Table 1:** Demographic, clinical and biomarker information of the MS cohort.

	**Baseline**	**Follow-up**
**(A) Demographic characteristics**	*n* = 312	*n* = 242
Female %	70.5	69.4
Age, mean years (*SD, range*)	42.9 (9.9, 19–68)	45.1 (9.8, 21–70)
Disease duration, mean years (*SD, range*)	11.0 (8.2, 0–43)	13.5 (8.5, 2–46)
Follow-up time, mean years (*SD, range*)		1.97 (0.34, 0.7–3.3)
Age at first symptom, mean years (*SD, range*)	31.4 (8.9, 7–56)	
*Centre (included patients)*		
Barcelona % (*n*)	18 (59)	18 (44)
Oslo % (*n*)	30 (95)	33 (78)
Berlin % (*n*)	22 (70)	18 (44)
Genova % (*n*)	28 (88)	31 (76)
**(B) Disease modifying treatment**	*n* = 311	*n* = 235
No treatment % (*n*)	30 (94)	26 (61)
Effective treatment % (*n*)	44 (138)	37 (87)
Highly-effective treatment % (*n*)	26 (79)	37 (87)
**(C) Clinical evaluation and biomarkers**		
*Multiple sclerosis classification*		
CIS % (*n*)	1.6 (5)	1.7 (4)
RRMS % (*n*)	81.4 (254)	78.5 (190)
SPMS % (*n*)	8.0 (25)	10.3 (25)
PPMS % (*n*)	9.0 (28)	9.5 (23)
*Neurological disability*		*n* = 275
EDSS, median (*IQR, range*)	2.0 (1.5–3.5, 0.0–8.0)	2.0 (1.0–3.5, 0.0–8.0)
Δ EDSS improving, % (*n*)		19 (51)
Δ EDSS stable, % (*n*)		65 (181)
Δ EDSS worsening, % (*n*)		16 (43)
T2 lesion volume ml, mean (*SD, range*)	8.5 (10.9, 0.1–64.5)	9.6 (11.1, 0.1–57)
Δ T2 lesion volume ml, mean (SD, range)		−0.4 (6.5, −52.2–53.7)
Normalized brain volume ml, mean (*SD, range*)	1507 (90, 1263–1724)	1453 (70, 1244–1666)
Serum neurofilament light levels, mean pg/ml (*SD, range*)	8.9 (7.0, 2.2–93.2)	8.7 (5.5, 2.3–45.5)

CIS = clinically isolated syndrome; EDSS = Expanded Disability Status Scale; IQR = interquartile range; PPMS = primary-progressive multiple sclerosis; RRMS = relapsing-remitting multiple sclerosis; SD = standard deviation; SPMS = secondary-progressive multiple sclerosis.

Inclusion criteria included age 18–80 years, CIS or MS diagnosis according to the 2010 McDonald’s criteria (Polman et al., 2011). Exclusion criteria were use of corticosteroids the last 30 days or a relapse in the month prior to inclusion, subjects not eligible for a blood draw, chronic diseases other than MS, and pregnancy during the course of the study. For patients previously treated with disease modifying treatments (DMT), a washout of at least three months was required (six months for ocrelizumab/rituximab; one year following alemtuzumab). Patients on DMTs were also included and needed to be stable for at least one year when treated with interferon (IFN)-beta or glatiramer acetate (GA) or at least six months for other treatments. We regarded highly-effective DMTs as alemtuzumab, natalizumab, rituximab, ocrelizumab and fingolimod, while the remaining DMTs were regarded as effective DMTs. Serum samples from age-and sex-matched healthy controls (HC) (Supplementary Table 1) were collected from the same four European MS centres (59 at baseline and 30 at follow-up). EDSS worsening at follow-up was defined as an increase of at least 1.0 for EDSS scores between 0.0 and 5.0, and an increase of at least 0.5 for EDSS levels of 5.5 and above. EDSS was defined as improved at follow-up if there was a reduced EDSS score of at least 1.0 for EDSS levels between 0.0 and 5.0, and at least 0.5 for scores above 5.0. Stable EDSS at follow-up was defined as EDSS levels including and below 5.0, with either the same EDSS level at follow-up or a change of just 0.5.

### 2.2 Standard protocol approvals, registrations, and patient consents

The Sys4MS project was approved by the IRBs of University of Genoa, Charité-Universitaetsmedizin, Hospital Clinic of Barcelona and the regional ethics committee (REC) in Norway for the University of Oslo (REC ID: 2011/1846 A). Patients provided signed informed consent prior to their enrolment on the study according to the Declaration of Helsinki.

### 2.3 Serum neurofilament light analysis

Serum samples were collected with 4 mL Vacuette Z Serum Clot Activator Tube® (Greiner bio-one International) and processed within one hour by centrifugation at 2000*g* for 10 min at 4 °C. Serum aliquots were immediately stored at -80 °C until analysis. Samples were thawed only once during the processing. Measurement of serum NfL samples was performed in the Clinical Neurochemistry Laboratory at the Sahlgrenska University Hospital, Sweden, by board certified laboratory technicians blind to clinical data, using an ultrasensitive Single molecule array (Simoa) assay described elsewhere (Disanto et al., 2017). A single batch of reagents was used; intra-assay coefficients of variation were below 10 % for all analyses. Two QC samples were run in duplicates in the beginning and the end of each run, repeatability and intermediate precision were 9.0% at 25 pg/mL and 5.2% at 80 pg/mL.

### 2.4 MRI acquisition

Images were acquired at different European MS centres. From Centre 1 (Barcelona), a 3D magnetization prepared rapid gradient echo (MPRAGE) sequence (0.86 × 0.86 × 0.86 mm resolution, repetition time (TR) = 1970 ms, echo time (TE) = 2.41 ms, inversion time (TI) = 1050 ms), an axial T1-weighted post-gadolinium contrast agent sequence (1 × 1 × 3 mm resolution, TR = 390 ms, TE = 2.65 ms), and a 3D fluid-attenuated inversion recovery (FLAIR) sequence (1 × 1 × 1 mm resolution, TR = 5000 ms, TE = 393 ms, TI = 1800 ms) were acquired longitudinally using a Tim Trio MRI (Siemens Medical Systems, Erlangen, Germany), and MAGNETOM Prisma MRI (Siemens Medical Systems) at the follow-up assessment from January 15th 2018. From Centre 2 (Oslo), a 3D sagittal brain volume (BRAVO) sequence for pre- and post-gadolinium contrast agent administration (1 × 1 × 1 mm resolution, TR = 8.16 ms, TE = 3.18 ms, TI = 450 ms, flip angle (FA) = 12°), and a 3D FLAIR sequence (1 × 1 × 1.2 mm resolution, TR = 8000 ms, TE = 127.25 ms, TI = 2240 ms) were acquired longitudinally using a Discovery MR750 MRI (GE Medical Systems). From Centre 3 (Berlin), a 3D sagittal MPRAGE sequence (1 × 1 × 1 mm resolution, TR = 1900 ms, TE = 3.03 ms, TI = 900 ms), and a 3D FLAIR sequence (1 × 1 × 1 mm resolution, TR = 6000 ms, TE = 388 ms, TI = 2100 ms) were acquired longitudinally using a Tim Trio MRI (Siemens Medical Systems, Erlangen, Germany). From Centre 4 (Genova), a sagittal fast-spoiled gradient-echo (FSPGR) or MPRAGE sequence (1 × 1 × 1 mm resolution, TR = 7.31–2300 ms, TE = 3.00 – 2.96 ms), a 3D turbo field echo (TFE) sequence for post-gadolinium contrast agent administration (1 × 1 × 1 mm resolution, TR = 8.67 ms, TE = 4.00 ms), and a 3D FLAIR sequence (1 × 1 × 1 mm resolution, TR = 6000 ms, TE = 122.16–388 ms, 1872–2100 ms) were acquired longitudinally using a Signa HDxt MRI (GE Medical Systems) and Ingenia MRI (Philips Medical Systems) at baseline and MAGNETOM Prisma MRI (Siemens Medical Systems) for follow-up assessment. All sequences included the upper cervical cord, as to not affect the analyses of the brainstem due to missing coverage.

### 2.5 MRI pre- and post-processing at Berlin reading centre

Pre-processing included alignment to Montreal Neurological Institute (MNI) – 152 standard space (using fslreorient2std), white and grey matter brain masking (using Computational Anatomy Toolbox 12 Toolbox for MATLAB) (Ashburner and Friston, 2005), N4-bias field correction (Advanced Normalization Tools, https://stnava.github.io/ANTs/) and linear, rigid body registration of T2-weighted (FLAIR) images to T1-weighted (MPRAGE, BRAVO, and FSPGR) images (FSL FLIRT) (Jenkinson et al., 2002, Jenkinson and Smith, 2001). First, MPRAGE scans were brain extracted and a transformation matrix was created using rigid body registration (6 degrees of freedom) of the individual brain extracted MPRAGE images to MNI-152 space with FSL FLIRT (Jenkinson et al., 2002, Jenkinson and Smith, 2001). The transformation matrix was applied to the native MPRAGE using spline interpolation, giving an MNI-standard space MPRAGE. Next, we obtained the FLAIR to the MNI-standard space transformation matrix, as with the MPRAGE. Then, we combined the FLAIR to MNI matrix with MPRAGE to MNI matrix using convert_xfm, and applied the combined matrix on the native FLAIR image using FLIRT. Lesion masks were then manually segmented using these coregistered MNI-standard space FLAIR images for each patient with ITK-SNAP. T1-weighted and FLAIR follow-up scans were co-registered to the individual first session using the transformation matrices saved from the first session transformation from native space images to MNI-152 standard space using FSL FLIRT. Post-contrast agent T1-weighted images were also co-registered to MNI-152 standard space and longitudinally when available.

T2-hyperintense lesion segmentation was performed using a semi-automated pipeline at one centre on co-registered T1-weighted images and T2-weighted FLAIR images by two experienced MRI technicians. Lesion segmentation was performed by two MRI technicians (10 and 12 years of experience in MS lesion segmentation), with subsequent segmentation quality control by an experienced neuroradiologist. Lesions were segmented and saved as binary masks using ITK-SNAP (www.itksnap.org) (Yushkevich et al., 2006). First session lesion masks were subsequently overlayed onto the second session co-registered T1-weighted and FLAIR images for editing, to include any T2-hyperintense lesion changes (i.e., new lesions, enlarging lesions, or decreasing lesions) in the follow-up scans. Any discrepancies in co-registrations that were visible between sessions were corrected manually using the ITK-SNAP automated registration tool before follow-up lesion mask edits. Lesion counts and volumes were extracted from lesion masks using fslmaths (https://fsl.fmrib.ox.ac.uk/fsl/fslwiki/Fslutils). T2-hyperintense lesion masks were used to fill longitudinally co-registered T1-weighted images using the FSL lesion filling tool (https://fsl.fmrib.ox.ac.uk/fsl/fslwiki/lesion_filling), utilizing white matter masks created from the Computational Anatomy Toolbox for SPM12 (CAT12, https://www.neuro.uni-jena.de/cat/). Lesion-filled T1-weighted images were then used for whole-brain white and grey matter volume extraction, including the follow-up session percent brain tissue volume change (PBVC), normalised for subject head size, was estimated with SIENA/X (Smith et al., 2002), part of FSL (Smith et al., 2004).

### 2.6 Disconnectome maps

Disconnectome maps were calculated using BCBtoolkit (Foulon et al., 2018). This approach uses diffusion-weighted imaging data from 10 healthy controls derived from the 7T tractography made available in de Schotten et al., Nat Commun, 2020 to track fibers passing through each lesion (Rojkova et al., 2016, Thiebaut de Schotten et al., 2011). It has been demonstrated that including only the provided 10 healthy controls when using BCBtoolkit to produce disconnectome maps matches the overall population well, resulting in maps with very high anatomical similarity across the lifespan (Foulon et al., 2018). Patients' lesions in the MNI152 space were registered to each control native space using affine and diffeomorphic deformations and subsequently used as seed for the tractography in Trackvis (Avants et al., 2011, Klein et al., 2009, Wang et al., 2007). The resulting tractograms were transformed to intermediate visitation maps, binarised, and brought to MNI152 space using the inverse of model deformations (Thiebaut de Schotten et al., 2015). Visitation maps are 3D representations of the brain in which the voxel values represent the number of streamlines from the probabilistic tractography with the lesion maps as seed voxels projecting through or “visiting” each voxel. The tractograms reported the track fiber as compromised or not in a binarised form. Finally, a percentage overlap map was computed by summing at each voxel in MNI space the normalized visitation map of each healthy subject. The white matter mask was made by including all non-zero values across the individual disconnectome maps and subsequently used in voxel-wise and global disconnectome analysis.

### 2.7 Global and regional disconnectome

Global disconnectome was calculated for each patient by computing the average disconnectome score across all white matter voxels. In the disconnectome map, the value in each voxel considers the interindividual variability of tract reconstructions in the training set comprising healthy controls and indicate a probability of disconnection from 0 to 100% for a given lesion (Thiebaut de Schotten et al., 2015). We thresholded the resulting map at >50%, indicating that at least half of the healthy controls have trajectories that intersect the lesion location in each patient for the corresponding fibers to be part of the patients’ disconnectome maps. Voxel-wise analysis was performed using FSL randomise (Winkler et al., 2014) on disconnectome maps from all patients at inclusion. The general linear model (GLM) applied to randomise was designed with a single-group average disconnectome map as response variable with serum NfL as explanatory variable, and with age, sex, disease duration, diagnosis, and treatment as additional covariates in the main design matrix. All categorical variables were transformed into dummy variables, in which diagnosis and treatment where combined to represent all respective conditions as unique explanatory variables. Combinations that did not occur in the sample were dropped from the GLM. Permutation-based inference was performed across 5000 iterations for both contrasts (positive and negative associations with serum NfL) with threshold-free cluster enhancement (TFCE) (Smith and Nichols, 2009). The resulting voxel-wise analysis was controlled for multiple testing across space using family-wise error rate (FWER) corrected t-stat map with a threshold of p = 0.05 (two-tailed).

### 2.8 Statistical analysis

Analyses were performed using R 4.0.3 (R Core Team, 2017). Continuous variables were normalized by subtracting the mean of each variable and dividing by the standard deviation using the generic scale-function in R. Linear regression analysis were also conducted on baseline measures of GD and T2LV at inclusion using the lm function in R (R Core Team, 2017). To investigate longitudinal associations between serum NfL and GD and T2LV from inclusion to two-year follow-up, two separate rLMM were conducted examining GD or T2LV as dependent variables. We used rlmer from the robustlmm R package (Koller, 2016), to model our longitudinal data, to better include extreme values than conventional LMMs. A conventional LMM was also performed using lmer from the lme4-package (Bates et al., 2015) to check for discrepancies between the model outputs of robust and conventional LMMs. All models included serum NfL, disease duration, age, sex, diagnosis, timepoint of visit, treatment as fixed effect terms, center, and subject identifier (ID) as random effects terms to account for variable intercept. Identical models where then conducted with T2LV as dependent variable.

### 2.9 Data availability

Anonymized summary data is available through the MultipleMS EU project and database (www.multiplems.eu) upon registration.

## 3 Results

### 3.1 Participant demographics and characteristics

At baseline, the MS cohort consisted of 71 % women, 83 % CIS and relapsing-remitting MS (RRMS) patients, with a mean age of 42.9 years (Table 1). The mean disease duration was 11 years and 30 % of the MS patients were untreated, while 44 % and 26 % were using effective and highly-effective DMTs, respectively. The MS subjects who completed follow-up were re-examined on average 2.0 years after baseline assessment (range = 0.7–3.3 years). At follow-up, 37 % of the MS subjects were using effective treatment, and 37% highly-effective DMTs, while the proportion of MS subjects currently not using DMTs was decreased to 26 %. For both time points, median EDSS was 2.0 (Interquartile range (IQR) = 1.5–3.5 at baseline and IQR = 1.0–3.5 at follow-up, range = 0.0–8.0 at both time points). Serum NfL levels at baseline were on average 8.9 pg/ml (SD ± 7.0 pg/ml) for those with MS and 7.0 pg/ml (SD ± 3.8) for HCs.

**Fig. 1:**
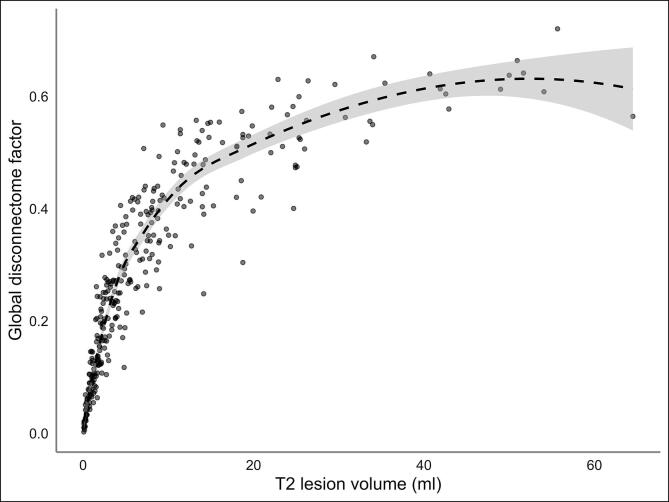
**Association between global disconnectome and T2 lesion volume**. Global disconnectome is shown as a factor between 0 and 1 for each individual disconnectome map averaged across all white matter voxels represented in the mask. The fit line represents the quadratic regression model based on the association between the global disconnectome factor and corresponding T2 lesion volume across all MS subjects.

The correlation between GD and T2LV was very high (r = 0.80, Fig. 1). At baseline serum NfL levels were significantly higher in patients with progressive MS (PMS), constituting both primary progressive MS (PPMS) and secondary progressive MS (SPMS) subjects, compared to HCs, after adjustment for age and sex. The serum NfL levels at follow-up were stable on a group-level for both the MS patients and the HCs as seen in Table 1 and Supplementary Table 1. The distributions of T2LV, EDSS and disease duration across the centers are shown in Supplementary Fig. 1.

### 3.2 Lesion and disconnectome maps

**Fig. 2:**
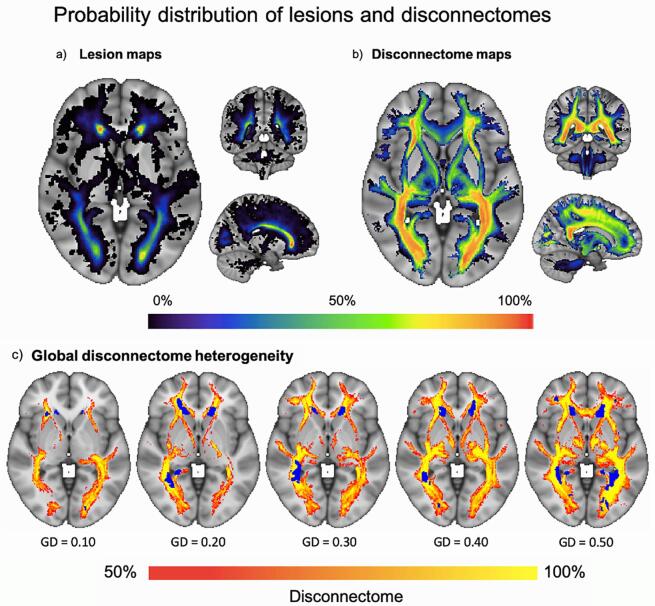
**Probability distribution heterogeneity of lesions and disconnectome in the MS sample.** An overview with representative example slices of the probability distribution of **(A)** lesion and **(B)** disconnectome maps, as seen in a large axial slice to the left and smaller coronal and sagittal slices on the top right and lower right, respectively. Masked out in both **(A)** and **(B)** are the borders of the resulting global masks and the colour-filled probability maps corresponding to the percentage of subjects with a lesion or disconnectome maps in the depict voxels. The corresponding colour bar is shown at the bottom of the figure. In **(C)** we depicted five subjects with different levels of GD, with blue colour highlighting the underlying lesion masks, while the red to yellow colour indicates the probability of disconnectivity. The GD value listed states the proportion and severity of white matter voxels affected, where 0.50 equals 50 %.

Fig. 2 shows a probabilistic map representing the overlap of lesions and disconnectome across all included subjects in addition to selected individual maps. The mean disconnectome maps across all individuals at both timepoints are depicted in Supplementary Fig. 2.

### 3.3 Cross-sectional multiple linear regression models

To investigate associations between sNfL and GD and T2LV at baseline, two separate multiple linear models were conducted, with GD and T2LV as dependent variables, respectively. Supplementary Table 2 summarizes the results from linear models testing for associations between GD and T2LV with serum NfL levels, different treatments, and MS phenotypes at baseline. Briefly, the model revealed significant associations between serum NfL and GD (t(2 8 3) = 3.11, std. beta = 0.14, CI = 0.05–0.23, p = 0.002), disease duration (t(2 8 3) = 4.46, std. beta = 0.28, CI = 0.16–0.40, p = 1.1 × 10^−5^). Significant effects were also evident for both DMT groups compared to no treatment, with effective treatment (t(2 8 3) = 2.70, std. beta = 0.35, CI = 0.10–0.61, p = 0.007) and highly-effective treatment (t(2 8 3) = 3.62, std. beta = 0.52, CI = 0.24–0.81, p = 3.4 × 10^−4^). In addition, effects of center was found where Genoa was associated with higher levels of brain dysconnectivity relative to Barcelona (t(2 8 3) = 2.38, std. beta = 0.35, CI = 0.06–0.64, p = 0.018). The T2LV models revealed significant positive effects of disease duration (t(2 8 3) = 2.89, std. beta = 0.14, CI = 0.01–0.27, p = 0.03), and sex (t(2 8 3) = 1.99, std. beta = 0.23, CI = 0.00–0.46, p = 0.047) whereby being female was associated with larger lesion volume. This model also revealed larger T2LV among subjects from Genoa relative to Barcelona (t(2 8 3) = 2.84, std. beta = 0.16, CI = 0.14–0.75, p = 0.005).

### 3.4 Longitudinal analyses with robust linear mixed models

**Fig. 3:**
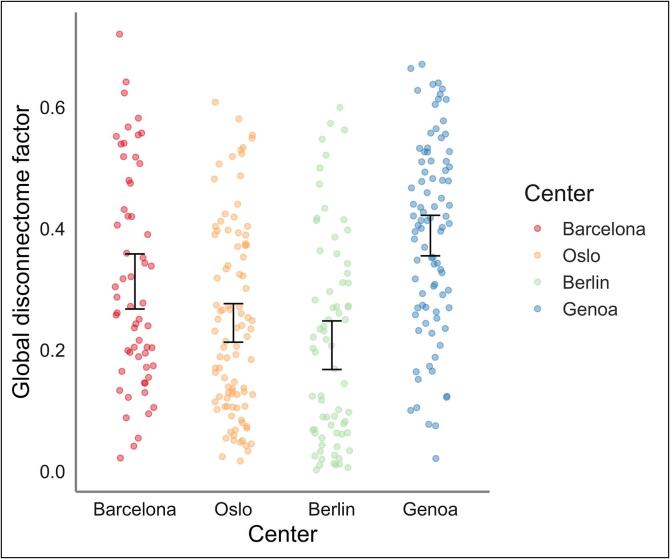
**An overview of the distribution of global disconnectome (GD) at baseline across the four centers.** As depicted for each individual, patients from Berlin had significantly lower GD (mean = 0.21, SD = 0.17), while patients from Genoa had significantly higher GD (mean = 0.39, SD = 0.16) relative to patients from Barcelona (mean = 0.31, SD = 0.17). GD in patients from Oslo (mean = 0.25, SD = 0.16) did not significantly differ from patients in Barcelona.

Table 2 summarize the results from the rLMM testing for associations between GD or T2LV with serum NFL, timepoint, disease duration, age, sex, treatment, center and MS phenotype as covariates. Patients recruited in Berlin had significantly lower global disconnectome (t = -2.19, std. beta = −0.41, CI = -0.77 to −0.04, p = 0.029), while subjects investigated in Genoa had a significant higher global disconnectome (t = 2.79, std. beta = 0.44, CI = 0.13–0.75, p = 0.005) relative to subjects from Barcelona as seen in Fig. 3. In addition, we also used the same set-up with centre while restricting the sample to RRMS subjects only (Supplementary Table 3).

**Table 2:** Robust linear mixed models testing for associations of GD and T2LV with serum NfL, including center as fixed effect term.

	**Global disconnectome**					**T2 lesion volume**				
*Predictors*	Std. β	*SE*	*CI*	*t*	*p*	Std. β	*SE*	*CI*	*t*	*p*
(Intercept)	−0.60	0.51	−1.60 – 0.41	−1.16	0.246	−0.53	0.32	−1.16 – 0.10	−1.64	0.101
Serum NfL	0.03	0.01	0.01 – 0.05	2.84	0.005	−0.01	0.01	−0.02 – 0.01	−0.82	0.414
Timepoint	−0.04	0.01	−0.06 – -0.01	−2.49	0.013	−0.00	0.01	−0.02 – 0.01	−0.53	0.596
Disease duration	0.14	0.06	0.02 – 0.25	2.35	0.019	0.10	0.03	0.03 – 0.16	2.81	0.005
Age	0.18	0.06	0.06 – 0.31	2.82	0.005	0.15	0.04	0.07 – 0.23	3.82	1.3 **× 10**^−^**^4^**
Sex [Female]	0.08	0.12	−0.16 – 0.32	0.63	0.528	0.09	0.08	−0.07 – 0.24	1.11	0.265
Phenotype [PMS]	0.62	0.49	−0.34 – 1.59	1.26	0.208	0.16	0.31	−0.44 – 0.77	0.53	0.600
Phenotype [RRMS]	0.58	0.49	−0.38 – 1.54	1.18	0.238	0.18	0.31	−0.42 – 0.79	0.59	0.554
Treatment [Effective]	0.01	0.02	−0.02 – 0.04	0.71	0.476	−0.01	0.01	−0.03 – 0.00	−1.47	0.141
Treatment [Highly effective]	0.01	0.01	−0.02 – 0.04	0.61	0.539	0.00	0.01	−0.01 – 0.02	0.10	0.922
Center [Oslo]	−0.29	0.16	−0.60 – 0.02	−1.82	0.068	−0.03	0.10	−0.23 – 0.16	−0.35	0.726
Center [Berlin]	−0.41	0.19	−0.77 – -0.04	−2.19	0.029	−0.04	0.12	−0.27 – 0.18	−0.38	0.703
Center [Genoa]	0.44	0.16	0.13 – 0.75	2.79	0.005	0.48	0.10	0.28 – 0.67	4.80	1.6 **× 10**^−^**^6^**
Serum NfL * Timepoint	−0.01	0.01	−0.03 to −0.00	−2.21	0.027	0.01	0.00	0.00 – 0.02	1.97	0.049
**Random Effects**										
σ^2^	0.00					0.00				
τ_00_	0.84 _ID_					0.33 _ID_				
ICC	1.00					1.00				
N	297 _ID_					297 _ID_				
Observations	506					505				
Marginal R^2^ / Conditional R^2^	0.236 / 0.998					0.267 / 0.999

Briefly, the GD model revealed that higher degree of disconnectivity was associated with higher serum NfL (t = 2.84, std. beta = 0.03, CI = 0.01–0.05, p = 0.005), longer disease duration (t = 2.35, std. beta = 0.14, CI = 0.02–0.25, p = 0.019), and higher age (t = 2.82, std. beta = 0.18, CI = 0.06–0.31, p = 0.005). We also observed a significant decrease in GD over time (t = -2.49, std. beta = -0.04, CI = -0.04- −0.01, p = 0.013). The T2LV model revealed that larger lesions volumes were associated with longer disease duration (t = 2.81, std. beta = 0.10, CI = 0.03–0.16, p = 0.005), and higher age (t = 3.82, std. beta = 0.15, CI = 0.07–0.23, p = 1.3 × 10^−4^). To assess longitudinal associations between changes in sNfL and GD, and between changes in sNfL and T2LV, from inclusion to 24-months follow-up, we added an interaction term with timepoint in the rLME models. The models revealed that the positive associations between sNfL and GD was reduced over time, and that the negative associations between sNfL and T2LV, albeit non-significant, became more negative over time (Table 2). Restricting the sample to RRMS subjects only did not alter our main results (Supplementary Table 3).

### 3.5 Higher probability of disconnectome in cerebellum and brainstem with increased serum NFL

**Fig. 4:**
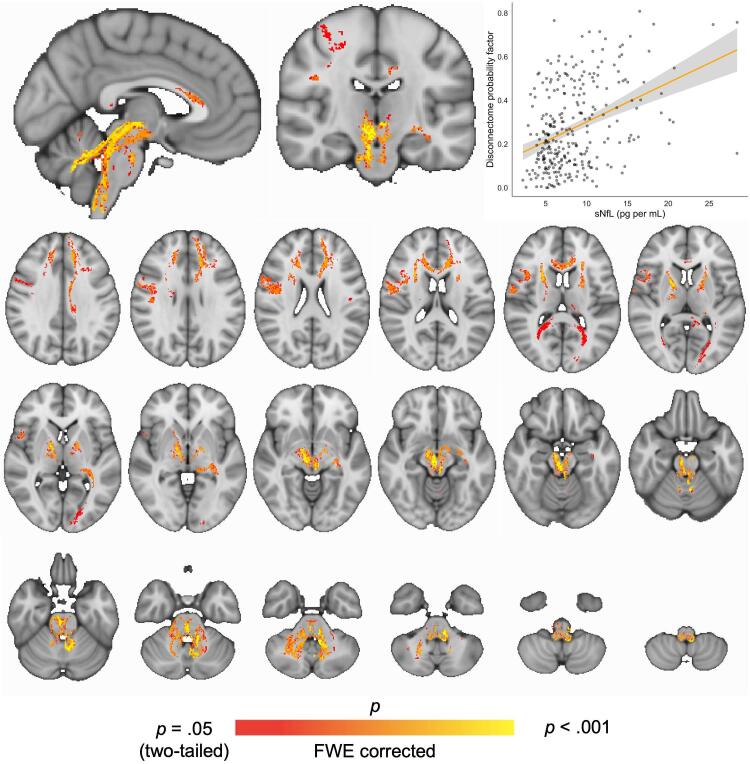
**Voxel-wise associations between disconnectome maps and serum NfL***.* Regional effects of serum NfL on brain disconnectome was found in cerebellum and brainstem using FSL randomise with TFCE. Age, sex, disease duration, diagnosis and treatment were included in the model. FWER-corrected t-stat map with threshold at p = 0.05 showing significant voxels in cerebellum and brainstem associated with increased serum NfL.

Fig. 4 shows the results from voxel-wise analysis testing for associations between disconnectome and serum NfL. TFCE and permutation-based corrections revealed significant effects in the cerebellar white matter and brainstem region, extending into right premotor cortices and frontal areas including forceps minor and anterior corona radiata.

To assess a potential confounding effect of disease duration on the association of disconnectivity in the cerebellum and brainstem and serum NfL, we added disease duration as a explanatory variable in our GLM. This did not affect the association between serum NfL and disconnectome. We also investigated the correlation between the significant voxels and disease duration at inclusion (Supplementary Fig. 3).

### 3.6 Comparing statistical output

Conventional linear mixed models (LMM) revealed similar results as the main robust linear mixed models (Supplementary Tables 4 and 5).

### 3.7 Outlier analysis

Outliers for serum NfL levels were removed from the GLM for the voxel-wise analysis if the serum NfL levels were higher than three standard deviations above the mean (n = 4 patients). The same approach was used for detecting sNfL outliers in the conventional LMM which is illustrated in Supplementary Figure 4 and 5. Outliers were not removed from the rLMM as these models are specifically designed to handle extreme values (Koller, 2016).

## 4 Discussion

Incorporating complex microstructural brain connectome information based on 7T diffusion MRI tractography data from healthy controls, the current disconnectome approach, utilizing T2-lesion masks to create individual brain disconnectivity maps, has provided new insights into different brain disorders, yet the neuroaxonal correlates have not been investigated (Foulon et al., 2018, Thiebaut de Schotten et al., 2020, Ulrichsen et al., 2021). Here we provide a link between imaging-based and biologically-assessed axonal damage. Our findings demonstrate that in-vivo evaluation of network-level perturbations beyond conventional T2-lesions is sensitive to neuroaxonal processes in MS, which are not necessarily identified by means of clinical neuroradiological evaluations. Expanding the initial global associations with voxel-wise cross-sectional analyses revealed that the associations between the probability of disconnectivity and serum NfL were primarily distributed in the cerebellar and brainstem regions. While further investigations and replications are required, these findings indicate that lesions in and around white matter fibers projecting into the cerebellum and brainstem are associated with higher serum NfL levels.

Disease duration was significantly associated with the probability of disconnectomes in the cerebellum and brainstem, but did not account for the association between global disconnectome and serum NfL. Supporting a potential role of cerebellum in MS pathophysiology and symptoms, a recent study reported that ataxia was associated with both atrophy and decreased functional connectivity in cerebellum (Cordani et al., 2020). The cerebellum has been shown to have important clinical impact in MS, yet not been as thoroughly investigated as its supratentorial counterpart (Parmar et al., 2018, Weier et al., 2015). Furthermore, lower number of Purkinje cells has been found in demyelinated lesions in cerebellum from MS patients compared with healthy subjects, which could support the observed links between serum NfL levels and brain disconnectivity (Redondo et al., 2015). Recently, the brainstem has gained more interest in imaging research due to improved segmentation methods, and genetic overlaps between brainstem sub-volumes and brain disorders, including MS, have been reported (Elvsashagen et al., 2020). Also, NfL from the cerebrospinal fluid (CSF) were highly correlated to brain lesions in the infratentorial region in a recent study exploring MRI lesion location and its relationship with CSF NfL (Adams et al., 2022).

The current connectivity-based approach allowed us to expand the distribution of anomalies beyond the visible T2-hyperintense brain lesions in MS patients, which offered a sensitive measure of neuropathology and axonal damage. Tractography and tractometry-based approaches are rare in MS due to technical challenges in harmonizing data from different MRI scanners and in the post-processing pipeline (Chamberland et al., 2020, Ciccarelli et al., 2008, Lipp et al., 2020). By using BCBtoolkit, which adapts high-resolution normative 7 T diffusion data from healthy controls, we avoid complex post-processing imaging methods and most sensitive scanner-related confounding factors. Further, since the disconnectomes are defined using tractograms from a normative, validated and independent training set, the only required inputs are accurately defined lesion masks from each patient. This enables integration of studies employing various clinical scanning protocols and enabling large-scale, collaborative studies with the inclusion of many patient groups from which advanced MRI may not be available or feasible.

While demonstrating an association between the extent of brain disconnectivity and a neuropathological marker of axonal damage, our results have to be interpreted in the context of their limitations. The patients were enrolled from different clinical centres in a real-world setting, thus any information regarding the use of DMT are due to clinical decisions outside the scope of this paper. Also, we did not have access to detailed information concerning recent disease activity besides information on new lesions in the brain. The mean serum NfL level across all patients was relatively low, and the skewed distribution with few cases with high serum NfL levels might have affected the results. Due to the relatively low levels of serum NfL for the MS subjects, we had to restrict our HCs dataset to the same sNfL batch only, since other HCs collected for other projects showed different levels of sNfL due to probable confounding batch- and population effects. Also, the stability of the serum NfL at follow-up could be reflecting improved MS care, which was explored in detail based on the same material in a recent publication (Brune et al., 2022). Further, non-random attrition e.g., due to disease progression, could have resulted in a more stable longitudinal MS cohort. As for the normative training set, several built-in limitations and misconceptions can be introduced at all stages of the underlying tractography process (Schilling et al., 2019). The fact that several scanners were used in the image acquisition, also including changes during the baseline collection of MRI data at Genoa, is something to consider when interpreting our results. Although, we have experienced that lesion maps derived from MR images are less affected compared to other more sensitive measures such as volumetrics derived from structural MRI scans. As seen in Fig. 3, the resulting GD estimates are converging with what we see from the demographic and clinical distributions across the centers (Supplementary Fig. 1), supporting that the confounding effects of scanner is not affecting our results in a substantial way. Further methodological developments and longitudinal studies are warranted to investigate the clinical and cognitive relevance and predictive value of brain disconnectome mapping in MS. New imaging methods incorporating different characteristics of lesions and diffuse pathology in the normal appearing white matter could also potentially benefit this approach by more sensitive lesion characteristics (Chard et al., 2021).

## 5 Conclusions

In conclusion, by providing evidence for an association between imaging-based brain disconnectome mapping and a blood biomarker reflecting axonal damage in patients with MS, these findings establish a neuropathological correlate of brain white matter affection, extending beyond conventional lesion-based characteristics. While these results support the clinical relevance of advanced network-based imaging approaches in MS, both as a combined marker and in detailed voxel-wise analyses, further studies investigating the functional and potential clinical sensitivity are warranted.

## CRediT authorship contribution statement

**Henning H. Rise:** Conceptualization, Formal analysis, Data curation, Writing – original draft, Writing – review & editing, Visualization. **Synne Brune:** Conceptualization, Investigation, Writing – original draft, Writing – review & editing. **Claudia Chien:** Methodology, Formal analysis, Data curation, Writing – original draft, Writing – review & editing. **Tone Berge:** Conceptualization, Investigation, Writing – original draft, Writing – review & editing, Supervision, Project administration, Funding acquisition. **Steffan D. Bos:** Writing – original draft, Writing – review & editing. **Magí Andorrà:** Methodology, Data curation, Writing – review & editing. **Irene Pulido Valdeolivas:** Investigation, Data curation, Writing – review & editing. **Mona K. Beyer:** Writing – original draft, Writing – review & editing, Project administration. **Piotr Sowa:** Writing – original draft, Writing – review & editing. **Michael Scheel:** Methodology, Writing – original draft, Writing – review & editing. **Alexander U. Brandt:** Writing – original draft, Writing – review & editing. **Susanna Asseyer:** Investigation, Writing – review & editing. **Kaj Blennow:** Methodology, Formal analysis, Writing – review & editing. **Mads L. Pedersen:** Methodology, Writing – original draft, Writing – review & editing. **Henrik Zetterberg:** Methodology, Formal analysis, Writing – review & editing. **Michel Thiebaut de Schotten:** Methodology, Software, Writing – review & editing. **Maria Cellerino:** Investigation, Writing – review & editing. **Antonio Uccelli:** Resources, Writing – review & editing, Supervision, Project administration. **Friedemann Paul:** Resources, Writing – review & editing, Supervision, Project administration. **Pablo Villoslada:** Conceptualization, Methodology, Resources, Writing – original draft, Writing – review & editing, Supervision, Project administration, Funding acquisition. **Hanne F. Harbo:** Conceptualization, Resources, Writing – original draft, Writing – review & editing, Supervision, Project administration, Funding acquisition. **Lars T. Westlye:** Conceptualization, Resources, Writing – original draft, Writing – review & editing, Supervision. **Einar A. Høgestøl:** Conceptualization, Formal analysis, Investigation, Data curation, Writing – original draft, Writing – review & editing, Supervision, Project administration.

## Declaration of Competing Interest

The authors declare the following financial interests/personal relationships which may be considered as potential competing interests: Henning H. Rise reports no disclosures. Synne Brune has received honoraria for lecturing from Biogen and Novartis. Claudia Chien has received honoraria for lecturing from Bayer and research grants from Novartis. Tone Berge has received unrestricted research grants from Biogen and Sanofi-Genzyme. Steffan Daniel Bos reports no disclosures. Magi Andorrà is currently an employee of Roche, all the work in this paper is based in his previous work at IDIBAPS. He holds stock from Bionure Farma SL, Attune Neurosciences Inc and Goodgut SL. Irene Pulido-Valdeolivas I has received travel reimbursement from Roche Spain, Novartis and Genzyme-Sanofi, and she is founder and holds stock in Aura Robotics SL. She is employee at UCB Pharma since July 2020. Mona Beyer has received honoraria for lecturing from Novartis and Biogen Idec and served on the advisory board for Biogen. Piotr Sowa has received honoraria for lecturing and travel support from Merck. Michael Scheel has received funding unrelated to this work from German Research Foundation, Federal Ministry of Education and Research and Federal Ministry for Economic Affairs and Energy. He is holding patents for 3D printing of computed tomography models and is shareholder of PhantomX GmbH. Alexander Brandt is cofounder and shareholder of Nocturne GmbH and Motognosis GmbH. He is named as inventor on several patent applications and patents describing multiple sclerosis serum biomarkers, motion analysis and retinal image analysis. Susanna Asseyer received a conference grant from Celgene and honoraria for lecturing from Alexion, Bayer, and Roche. Kaj Blennow has served as a consultant, at advisory boards, or at data monitoring committees for Abcam, Axon, Biogen, JOMDD/Shimadzu. Julius Clinical, Lilly, MagQu, Novartis, Roche Diagnostics, and Siemens Healthineers, and is a co-founder of Brain Biomarker Solutions in Gothenburg AB (BBS), which is a part of the GU Ventures Incubator Program. Mads L. Pedersen reports no disclosures. Henrik Zetterberg has served at scientific advisory boards for Eisai, Denali, Roche Diagnostics, Wave, Samumed, Siemens Healthineers, Pinteon Therapeutics, Nervgen, AZTherapies and CogRx, has given lectures in symposia sponsored by Cellectricon, Fujirebio, Alzecure and Biogen, and is a co-founder of Brain Biomarker Solutions in Gothenburg AB (BBS), which is a part of the GU Ventures Incubator Program. Michel Thiebaut de Schotten reports no disclosures. Marie Cellerino reports no disclosures. Antonio Uccelli has received personal compensation from Novartis, Biogen, Merck, Roche and Sanofi Genzyme for public speaking and advisory boards. AU received funding for research by Fondazione Italiana Sclerosi Multipla, the Italian Ministry of Health and Banco San Paolo. Friedemann Paul received honoraria and research support from Alexion, Bayer, Biogen, Chugai, MerckSerono, Novartis, Genyzme, MedImmune, Shire, Teva, and serves on scientific advisory boards for Alexion, MedImmune and Novartis. He has received funding from Deutsche Forschungsgemeinschaft (DFG Exc 257), Bundesministerium für Bildung und Forschung (Competence Network Multiple Sclerosis), Guthy Jackson Charitable Foundation, EU Framework Program 7, National Multiple Sclerosis Society of the USA. Pablo Villoslada received consultancy fees and hold stocks from Accure Therapeutics SL, Spiral Therapeutics Inc, Clight Inc, Neuroprex Inc, QMenta Inc and Attune Neurosciences Inc. Hanne F. Harbo has received travel support, honoraria for advice or lecturing from Biogen Idec, Sanofi-Genzyme, Merck, Novartis, Roche, and Teva and an unrestricted research grant from Novartis. Lars T. Westlye reports no disclosures. Einar Høgestøl received honoraria for lecturing and advisory board activity from Biogen, Merck and Sanofi-Genzyme and unrestricted research grant from Merck.
