# Supplementary material for: Brain disconnectome mapping derived from white matter lesions and serum neurofilament light levels in multiple sclerosis: A longitudinal multicenter study
Source: Neuroimage Clin. 2022 Jun 25;35:103099. doi: 10.1016/j.nicl.2022.103099 (PMC9253471; doi:10.1016/j.nicl.2022.103099)
Supplement: Supplementary data 1 [file mmc1.pdf]

Supplementary material: Brain disconnectome in multiple sclerosis

Supplementary Table 1 Demographic characteristics and serum NfL in the healthy control group

	Baseline	Follow-up
	n = 59	n = 30
Female % (n)	80 (47)	83 (25)
Age, mean years (SD, range)	39.9 (11.8, 22-71)	41.2 (12.5, 26-73)
Serum Neurofilament Light levels, mean pg/mL (SD, range)	7.0 (3.8, 2.2-21.6)	7.8 (3.7, 3.0-16.2)

SD, standard deviation

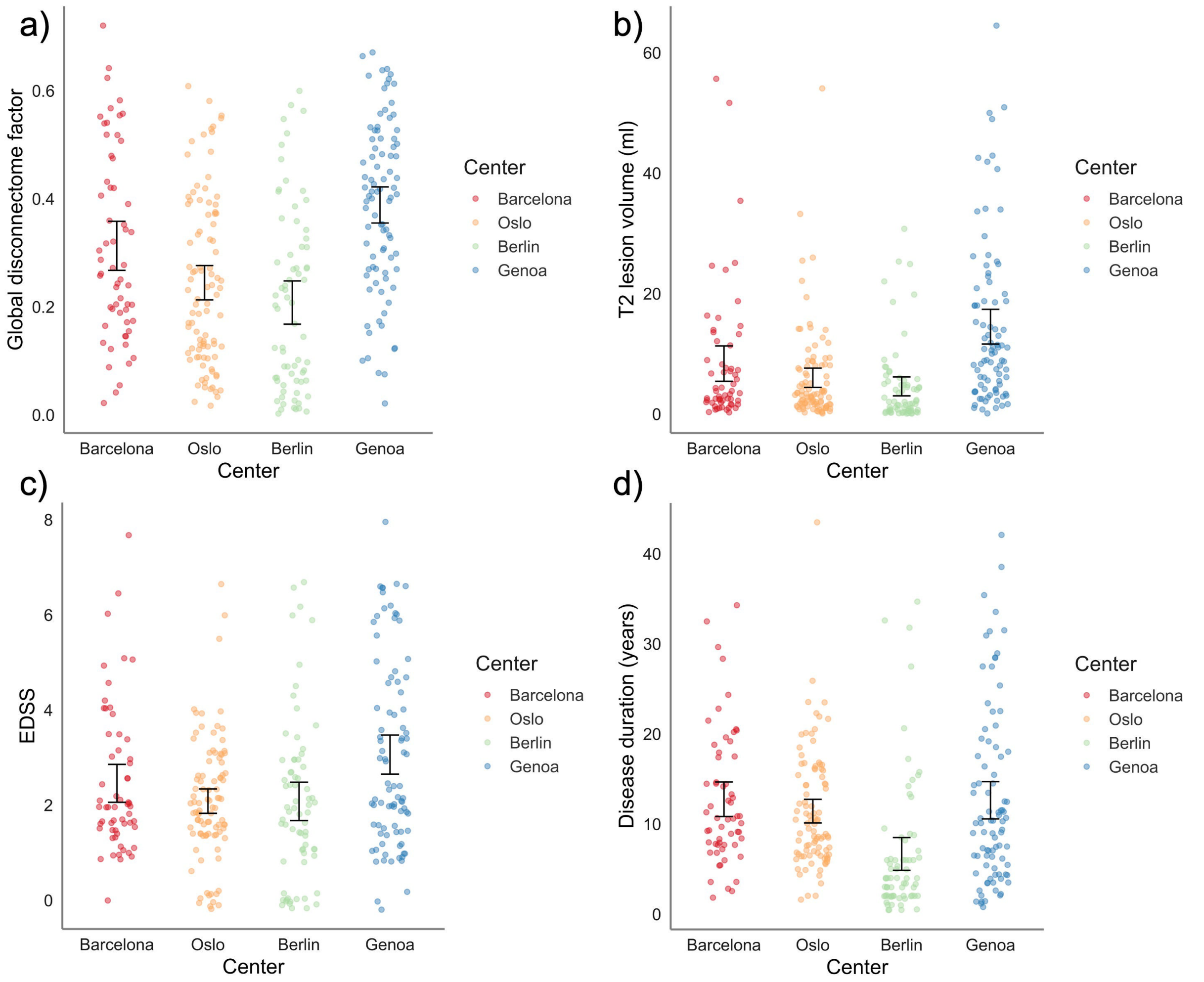

Supplementary Figure 1 An overview of the distribution of a) global disconnectome, b) T2 lesion volume, c) EDSS, and d) disease duration across the four centers.

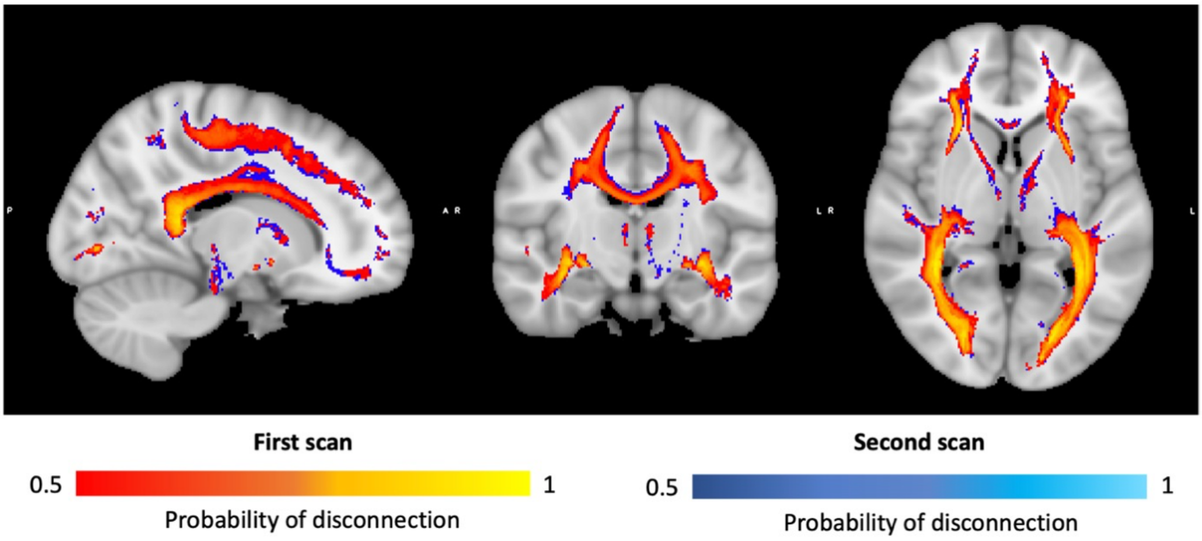

Supplementary Figure 2 An overview of the summarized global disconnectome maps at both timepoint. With the probability of disconnection, ranging from 0.5 to 1, shown in red gradually changing to yellow for the first timepoint and for dark blue gradually changing to light blue for the second timepoint. The two maps are overlayed as to visualize the longitudinal change.

Cross-sectional analyses with linear regression models

To investigate associations between sNfL and GD and T2LV at baseline, two separate linear regression models were conducted, with GD and T2LV as dependent variables, respectively.

Supplementary Table 2 summarizes the results from linear models testing for associations between GD and T2LV with serum NfL levels, different treatments and MS phenotypes at baseline. Briefly, the model revealed significant associations between serum NfL and GD (t(286) = 4.62, CI = 0.08-0.21, p = 5.7 x 10^{-6}), age (t(286) = 3.90, CI = 0.12-0.36, p = 1.2 x 10^{-4}), and progressive MS phenotypes (t(286) = 3.94, CI = 0.70-2.19, p = 1.0 x 10^{-4}). Significant effects were also evident for both DMT groups compared to no treatment, with effective treatment (t(286) = 3.29, CI = 0.18-0.70, p = 0.001) and highly-effective treatment (t(286) = 4.75, CI = 0.43-1.04, p = 3.3 x 10^{-6}) being associated with higher levels of brain disconnectivity. The T2LV models revealed a significant association with serum NfL (t(286) = 2.89, CI = 0.02-0.09, p = 0.004). In addition, the use of any DMTs compared to no treatment was associated with larger lesion volume, for both effective treatment (t(286) = 2.71, CI = 0.05-0.29, p = 0.007), as well as highly-effective treatment (t(286) = 3.49, CI = 0.15-0.55, p = 0.001).

Supplementary Table 2 Linear regression for global disconnectome and lesion volume at baseline with serum NfL

	Global disconnectome					T2 lesion volume				
Predictors	*Std. β*	*SE*	*CI*	*t*	*p*	*Std. β*	*SE*	*CI*	*t*	*p*
(Intercept)	-0.71	0.46	-1.60 – 0.19	-1.55	0.123	-0.52	0.48	-1.46 – 0.42	-1.08	0.280
Serum NfL	0.14	0.05	0.05 – 0.23	3.11	**0.002**	0.09	0.05	-0.00 – 0.18	1.88	0.062
Disease duration	0.28	0.06	0.16 – 0.40	4.46	**1.1×10^{-5}**	0.14	0.07	0.01 – 0.27	2.18	**0.030**
Age	0.09	0.06	-0.04 – 0.22	1.36	0.176	0.12	0.07	-0.01 – 0.26	1.79	0.074
Sex [Female]	0.13	0.11	-0.09 – 0.35	1.16	0.246	0.23	0.12	0.00 – 0.46	1.99	**0.047**
Phenotype [PMS]	0.68	0.47	-0.23 – 1.60	1.47	0.143	0.65	0.49	-0.31 – 1.61	1.34	0.181
Phenotype [RRMS]	0.26	0.44	-0.61 – 1.14	0.60	0.551	-0.03	0.47	-0.95 – 0.89	-0.06	0.950
Treatment [Effective]	0.35	0.13	0.10 – 0.61	2.70	**0.007**	0.19	0.14	-0.08 – 0.45	1.37	0.173
Treatment [Highly effective]	0.52	0.14	0.24 – 0.81	3.62	**3.4×10^{-4}**	0.27	0.15	-0.03 – 0.57	1.79	0.074
Center [Oslo]	-0.21	0.14	-0.49 – 0.07	-1.45	0.148	-0.04	0.15	-0.34 – 0.25	-0.28	0.776
Center [Berlin]	-0.20	0.17	-0.54 – 0.13	-1.19	0.236	-0.06	0.18	-0.41 – 0.29	-0.33	0.743
Center [Genoa]	0.35	0.15	0.06 – 0.64	2.38	**0.018**	0.44	0.16	0.14 – 0.75	2.84	**0.005**
Observations	295				295					
R^2 / R^2 adjusted	0.344 / 0.318					0.284 / 0.256				

Sensitivity analysis

We used the same set-up as the main rLMM models, but restricting the sample to RRMS subjects only (Supplementary Table 3).

Supplementary Table 3 Robust linear mixed models predicting serum NfL with global disconnectome and lesion volume including centre as random effect term for RRMS subjects only

	Global disconnectome				T2 lesion volume			
Predictors	*β*	*CI*	*t*	*p*	*β*	*CI*	*t*	*p*
(Intercept)	-0.14	-0.64 – 0.37	-0.53	0.595	-0.30	-0.59 – -0.00	-1.98	**0.048**
Serum NfL	0.03	0.01 – 0.06	2.66	**0.008**	0.00	-0.02 – 0.02	0.03	0.976
Timepoint	-0.00	-0.01 – 0.01	-0.40	0.692	0.02	0.01 – 0.03	6.09	**<0.001**

Age	0.17	0.04 – 0.31	2.62	**0.009**	0.12	0.04 – 0.20	2.98	**0.003**
Sex [Female]	0.09	-0.20 – 0.39	0.62	0.535	0.12	-0.06 – 0.29	1.27	0.204
Treatment [Effective]	0.03	-0.01 – 0.06	1.43	0.152	-0.00	-0.02 – 0.02	-0.10	0.923
Treatment [Highly-effective]	0.05	0.01 – 0.08	2.32	**0.021**	0.03	0.01 – 0.05	2.50	**0.012**
Serum NfL * Timepoint	-0.02	-0.03 – -0.00	-2.20	**0.028**	0.00	-0.01 – 0.02	0.55	0.580
**Random Effects**								
σ^2	0.00				0.00			
τ_{00}	0.99 $_{ID}$				0.35 $_{ID}$			
	0.18 $_{center}$				0.06 $_{center}$			
ICC	1.00				1.00			
N	243 $_{ID}$				243 $_{ID}$			
	4 $_{center}$				4 $_{center}$			
Observations	412				411			
Marginal R^2 / Conditional R^2	0.027 / 0.998				0.039 / 0.998			

Comparing statistical output

Supplementary Table 4 Overview of the model performance of linear mixed models compared with robust linear mixed models for global disconnectome.

	**Global disconnectome Linear mixed model**				**Global disconnectome Robust linear mixed model**			
Predictors	*β*	*CI*	*t*	*p*	*β*	*CI*	*t*	*p*
(Intercept)	-0.59	-1.48 – 0.30	-1.29	0.197	-0.60	-1.60 – 0.41	-1.16	0.246
Serum NfL	0.07	0.02 – 0.12	2.64	**0.008**	0.03	0.01 – 0.05	2.84	**0.005**
Timepoint	-0.07	-0.11 – -0.03	-3.88	**1.1×10^{-4}**	-0.04	-0.06 – -0.01	-2.49	**0.013**
Disease duration	0.24	0.13 – 0.36	4.10	**4.1×10^{-5}**	0.14	0.02 – 0.25	2.35	**0.019**
Age	0.11	-0.01 – 0.22	1.77	0.076	0.18	0.06 – 0.31	2.82	**0.005**
Sex [Female]	0.12	-0.09 – 0.34	1.11	0.266	0.08	-0.16 – 0.32	0.63	0.528
Phenotype [PMS]	0.71	-0.16 – 1.59	1.61	0.108	0.62	-0.34 – 1.59	1.26	0.208
Phenotype [RRMS]	0.57	-0.29 – 1.42	1.30	0.193	0.58	-0.38 – 1.54	1.18	0.238
Treatment [Effective]	0.04	-0.03 – 0.12	1.09	0.274	0.01	-0.02 – 0.04	0.71	0.476
Treatment [Highly effective]	0.07	0.00 – 0.14	2.07	**0.038**	0.01	-0.02 – 0.04	0.61	0.539

Center [Oslo]	-0.30	-0.57 – -0.02	-2.10	**0.035**	-0.29	-0.60 – 0.02	-1.82	0.068
Center [Berlin]	-0.31	-0.64 – 0.01	-1.88	0.060	-0.41	-0.77 – -0.04	-2.19	**0.029**
Center [Genoa]	0.37	0.09 – 0.64	2.57	**0.010**	0.44	0.13 – 0.75	2.79	**0.005**
Serum NfL * Timepoint	-0.02	-0.05 – 0.01	-1.41	0.159	-0.01	-0.03 – -0.00	-2.21	**0.027**
**Random Effects**								
σ^2	0.01				0.00			
τ_{00}	0.68_{ID}				0.84_{ID}			
ICC	0.98				1.00			
N	297_{ID}				297_{ID}			
Observations	506				506			
Marginal R^2 / Conditional R^2	0.281 / 0.986				0.236 / 0.998			

Supplementary Table 5 Overview of the model performance of linear mixed models compared with robust linear mixed models for T2 lesion volume.

	**T2 lesion volume Linear mixed model**				**T2 lesion volume Robust linear mixed model**			
Predictors	*β*	*CI*	*t*	*p*	*β*	*CI*	*t*	*p*
(Intercept)	-0.45	-1.40 – 0.50	-0.93	0.354	-0.53	-1.16 – 0.10	-1.64	0.101
Serum NfL	-0.01	-0.03 – 0.02	-0.48	0.634	-0.01	-0.02 – 0.01	-0.82	0.414
Timepoint	-0.01	-0.04 – 0.02	-0.55	0.581	-0.00	-0.02 – 0.01	-0.53	0.596
Disease duration	0.11	0.00 – 0.22	2.01	**0.044**	0.10	0.03 – 0.16	2.81	**0.005**
Age	0.23	0.11 – 0.35	3.77	**1.6 x 10^{-4}**	0.15	0.07 – 0.23	3.82	**1.3 x 10^{-4}**
Sex [Female]	0.20	-0.03 – 0.43	1.70	0.088	0.09	-0.07 – 0.24	1.11	0.265
Phenotype [PMS]	0.24	-0.68 – 1.15	0.51	0.613	0.16	-0.44 – 0.77	0.53	0.600
Phenotype [RRMS]	0.20	-0.71 – 1.11	0.43	0.665	0.18	-0.42 – 0.79	0.59	0.554
Treatment [Effective]	-0.01	-0.04 – 0.03	-0.37	0.712	-0.01	-0.03 – 0.00	-1.47	0.141
Treatment [Highly effective]	0.01	-0.02 – 0.04	0.76	0.447	0.00	-0.01 – 0.02	0.10	0.922
Center [Oslo]	-0.13	-0.42 – 0.17	-0.85	0.397	-0.03	-0.23 – 0.16	-0.35	0.726
Center [Berlin]	-0.10	-0.45 – 0.24	-0.59	0.558	-0.04	-0.27 – 0.18	-0.38	0.703
Center [Genoa]	0.54	0.24 – 0.83	3.59	**3.4 x 10^{-4}**	0.48	0.28 – 0.67	4.80	**1.6 x 10^{-6}**
Serum NfL * Timepoint	0.01	-0.01 – 0.02	0.67	0.501	0.01	0.00 – 0.02	1.97	**0.049**
**Random Effects**								
σ^2	0.00				0.00			
τ_{00}	0.78_{ID}				0.33_{ID}			

ICC	1.00	1.00
N	297 ID	297 ID
Observations	505	505
Marginal R^2 / Conditional R^2	0.223 / 0.998	0.267 / 0.999

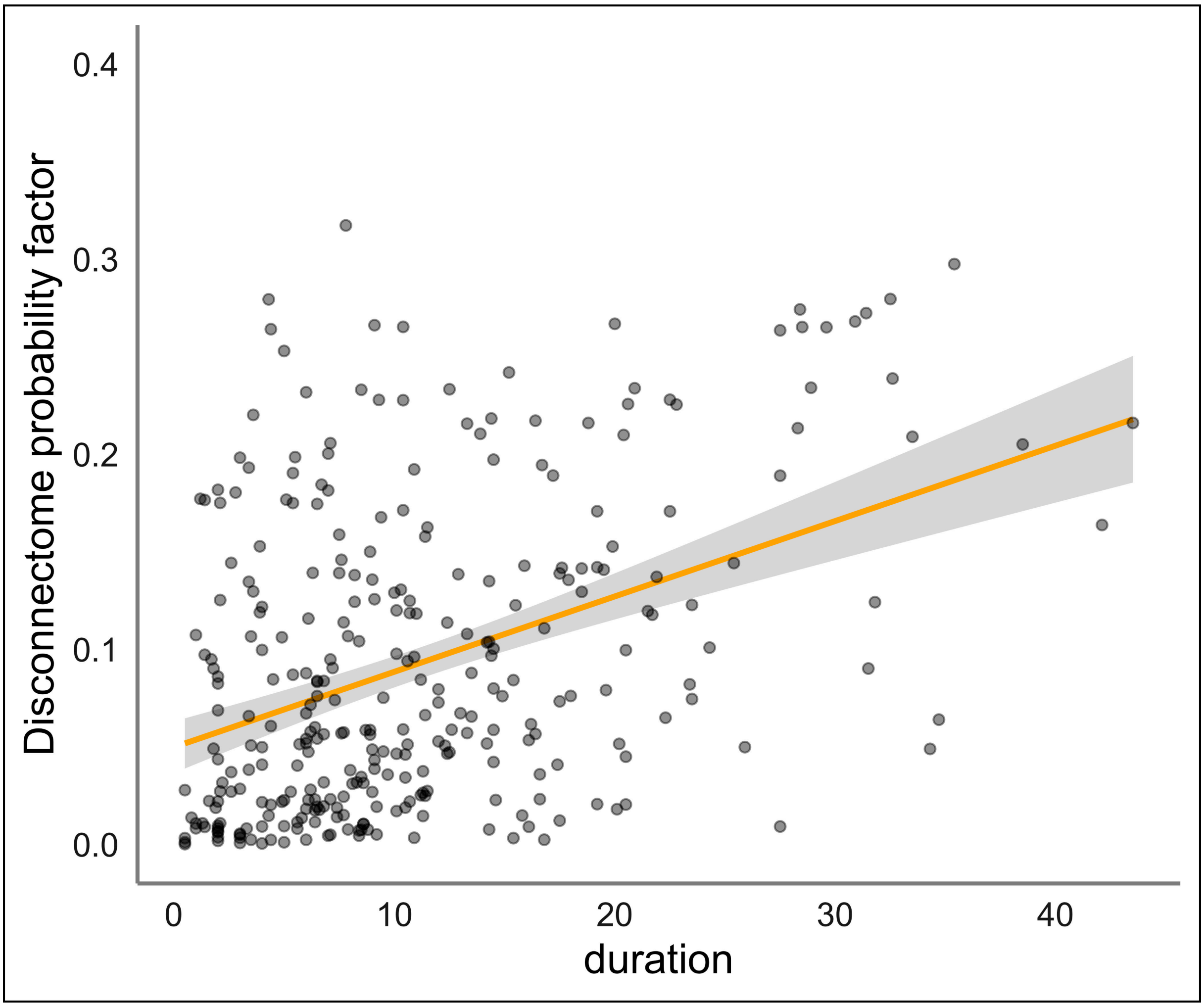

Supplementary Figure 3 Scatter plots visualizing the associations between the probability of a disconnectome in the significant voxels in of serum NfL levels across the complete sample with disease duration on the x-axis (std. beta = 0.01, p = 2.4 x 10^{-4}).

Outlier analysis

Outlier detection for serum NfL scores was performed following Tukey's fence method, where a score is considered an outlier if the value is either below the first quartile - 1.5 *

interquartile range (IQR) or above the third quartile + 1.5* IQR. However, removal of serum NfL outliers did not affect the overall results, as described in Supplementary Table 7.

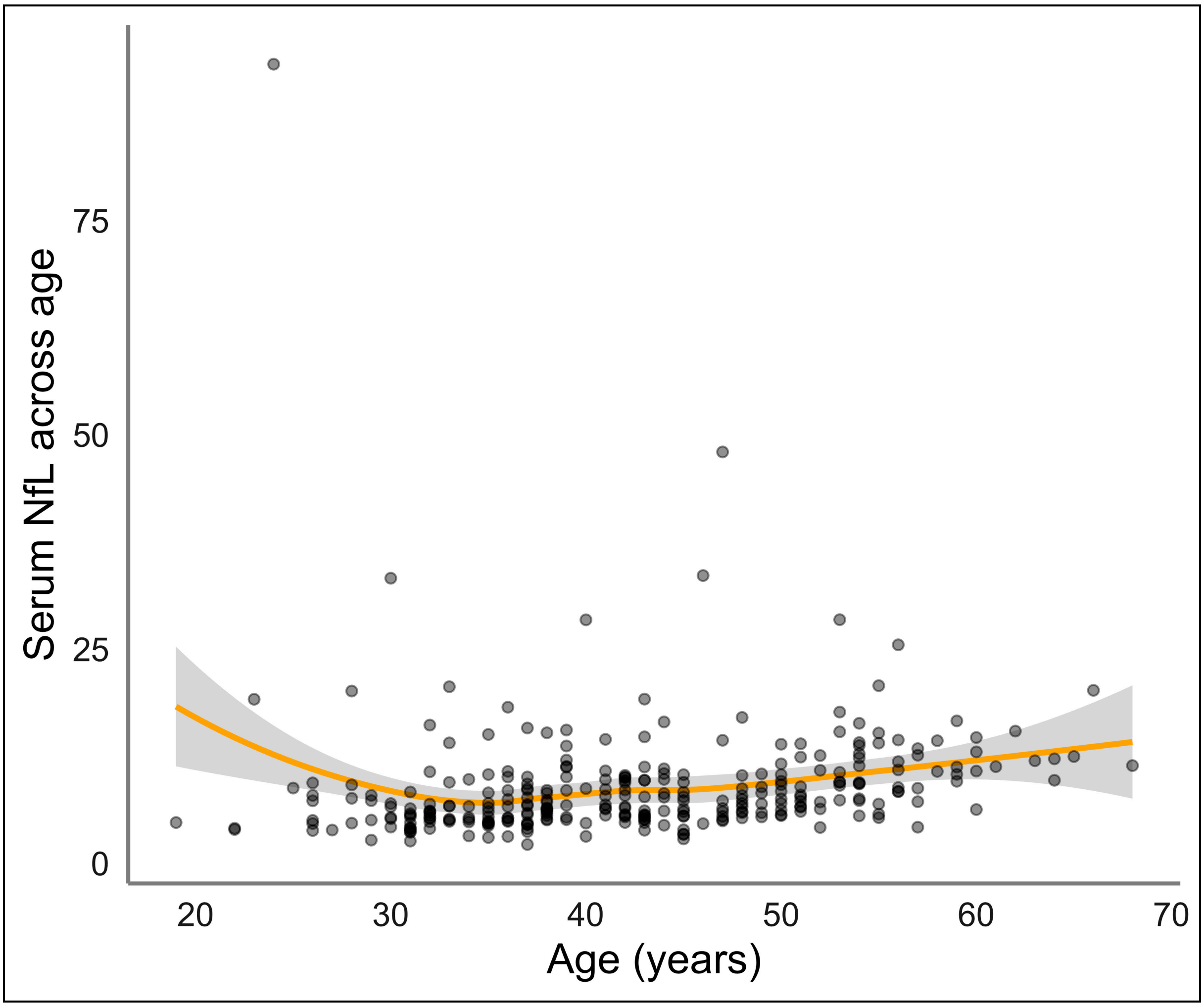

Supplementary Figure 4 Scatter plot visualizing the distributions of serum NfL levels across the complete sample with age on the x-axis.

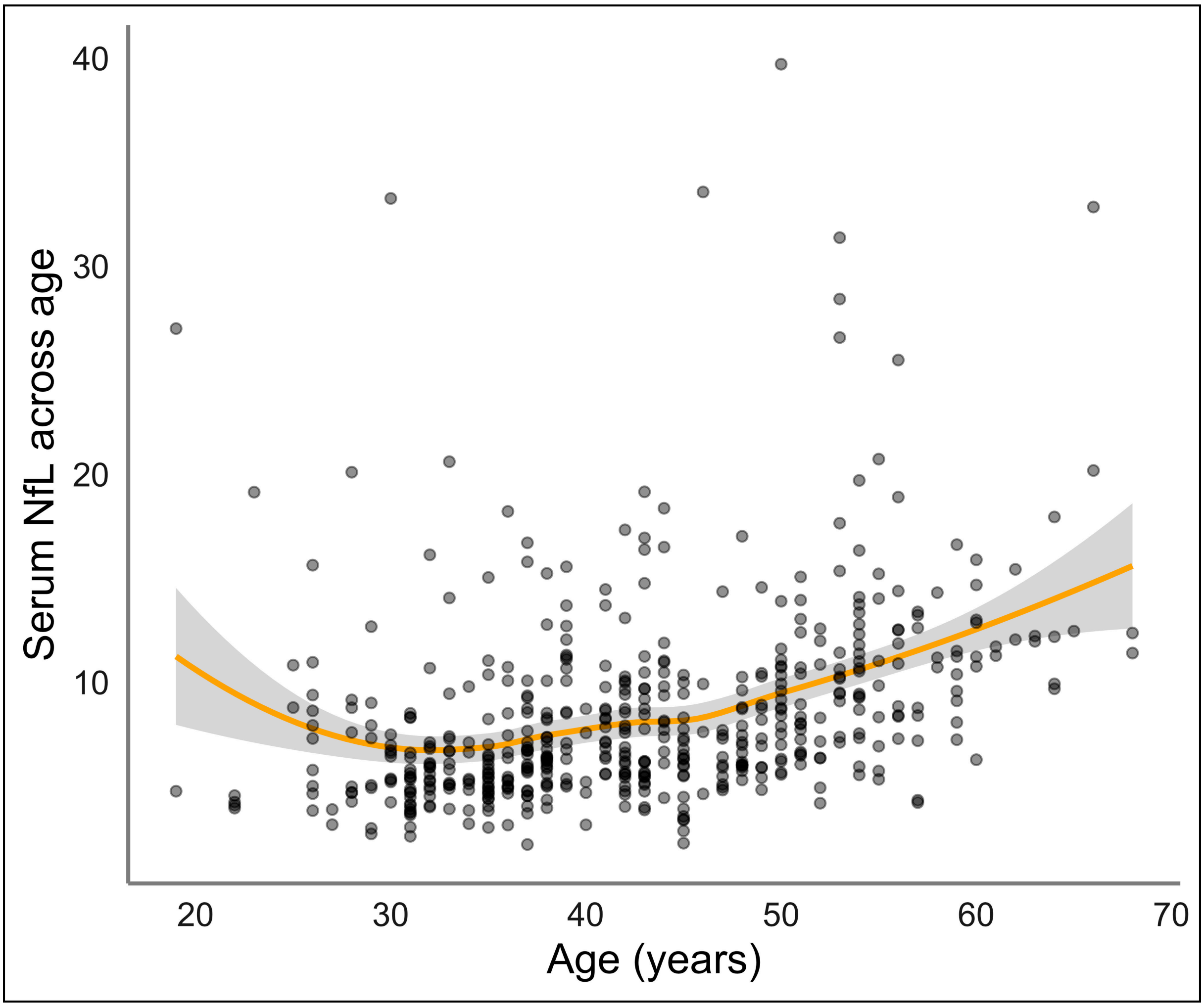

Supplementary Figure 5 Scatter plot visualizing the distributions of serum NfL levels across the sample with age on the x-axis, excluding outliers.
